# TLR4-Dependent Secretion by Hepatic Stellate Cells of the Neutrophil-Chemoattractant CXCL1 Mediates Liver Response to Gut Microbiota

**DOI:** 10.1371/journal.pone.0151063

**Published:** 2016-03-22

**Authors:** Amélie E. Bigorgne, Beena John, Mohammad R. Ebrahimkhani, Masami Shimizu-Albergine, Jean S. Campbell, Ian N. Crispe

**Affiliations:** 1 Seattle Biomedical Research Institute, 307 North Westlake Avenue, Seattle, Washington, 98109–5219, United States of America; 2 Department of Pathology, University of Washington, Seattle, Washington, 98195–7470, United States of America; Universite de Rennes 1, FRANCE

## Abstract

**Background & Aims:**

The gut microbiota significantly influences hepatic immunity. Little is known on the precise mechanism by which liver cells mediate recognition of gut microbes at steady state. Here we tested the hypothesis that a specific liver cell population was the sensor and we aimed at deciphering the mechanism by which the activation of TLR4 pathway would mediate liver response to gut microbiota.

**Methods:**

Using microarrays, we compared total liver gene expression in WT versus TLR4 deficient mice. We performed *in situ* localization of the major candidate protein, CXCL1. With an innovative technique based on cell sorting, we harvested enriched fractions of KCs, LSECs and HSCs from the same liver. The cytokine secretion profile was quantified in response to low levels of LPS (1ng/mL). Chemotactic activity of stellate cell-derived CXCL1 was assayed in vitro on neutrophils upon TLR4 activation.

**Results:**

TLR4 deficient liver had reduced levels of one unique chemokine, CXCL1 and subsequent decreased of neutrophil counts. Depletion of gut microbiota mimicked TLR4 deficient phenotype, i.e., decreased neutrophils counts in the liver. All liver cells were responsive to low levels of LPS, but hepatic stellate cells were the major source of chemotactic levels of CXCL1. Neutrophil migration towards secretory hepatic stellate cells required the TLR4 dependent secretion of CXCL1.

**Conclusions:**

Showing the specific activation of TLR4 and the secretion of one major functional chemokine—CXCL1, the homolog of human IL-8-, we elucidate a new mechanism in which Hepatic Stellate Cells play a central role in the recognition of gut microbes by the liver at steady state.

## Introduction

The unique microenvironment in the liver has been attributed to its close relationship to the gut.[1] The liver receives significant amounts of lipopolysaccharide (LPS) and other bacterial products from the intestine via the portal blood. Innate immunity is mediated through toll-like receptors (TLRs) with an elaborated crosstalk between liver parenchymal and non-parenchymal cells: Hepatocytes, Kupffer cells (KCs), Liver Sinusoidal Endothelial Cells (LSECs) and Hepatic Stellate Cells (HSCs) all express CD14 and TLR4, the co-receptors for LPS.[2, 3, 4] The recognition of commensal bacteria is required for intestinal homeostasis.[5] Microbiota interaction with liver cells has been studied in a number of chronic liver diseases such as alcoholic liver disease and non-alcoholic steatohepatitis.[6, 7, 8] Gut microbiota impacts liver immune function in diverse ways, which include inhibition of liver dendritic cells maturation[9] and hepatic fibrosis via TGF-beta1.[10] In our study, we investigate the effect of the microbiota on the abundance of liver neutrophils in the steady state liver.

We have previously shown in TLR4 deficient mice and using orthotopic mouse liver transplantation, that TLR4 plays a crucial role in the immune function of the liver.[11, 12]. Since the recognition of bacterial compounds by liver cells has important consequences for systemic immune homeostasis, it is important to identify which liver population plays the major role in this process. We based the present study on the hypothesis that, at steady state, the gut microbiota provides the main ligands for TLR4 in the liver. By depleting the commensal microbiota in wild type mice using an oral broad-spectrum antibiotic treatment, we reproduced the phenotype of LPS unresponsive TLR4 deficient mice and characterized the consequences for liver immune function.

We show that hepatic stellate cells plays a central role in sensing the gut microbiota, with a necessary activation of TLR4 and subsequent secretion of one specific chemokine, CXCL1, which promotes neutrophil recruitment. A recently identified mechanism is the release of extracellular chromatin fibers decorated with antimicrobial proteins, termed neutrophil extracellular traps (NETs), which target bacterial virulence factors.[13] Stimuli such as IL-8 (the structural CXCL1 homolog in human) and LPS prolong neutrophils lifespan and induce NETs efficiently.[14] The ability of NETs to directly prime T cells by reducing their activation threshold and consequently to enhance adaptive immune responses,[15] makes them a good candidate for the mechanism occurring in the liver and may explain why neutrophils are present.

Hepatic stellate cells—also named Ito cells or lipocytes—are located in the Space of Disse, exposed to a transudate of portal blood *via* the fenestrations in the liver sinusoidal endothelial cells. Besides their well-known role in liver fibrosis, there is current interest in their antigen-presenting potential.[16, 17] In addition, here we show that Hepatic Stellate Cells are central in the sensing of the gut microbiota and are capable of sending homing signals to neutrophils at steady state.

## Experimental Procedures

### Mice

C57BL/10ScNJ (TLR4 deficient) and C57BL/10SnJ (WT) mice were purchased from the Jackson Laboratory (Bar Harbor, ME) and housed in a specific pathogen-free environment in conformance with institutional guidelines for animal care. Animal welfare consisted in the inspection a minimum of x3 per week, and any found to be in distress were painlessly euthanized. For experiments, animals were euthanized using CO_2_, followed by dissection in a manner incompatible with survival that included severing the great vessels. The experiments were approved by the Institutional Animal Care and Use Committee, Seattle BioMed, WA, USA. All mice were used between 6–9 weeks of age.

### Affymetrix Gene chip hybridization

Total RNA was isolated from the frozen liver samples of WT and TLR4 deficient mice using TRIzol (Invitrogen) and its quality assessed with the Agilent bioanalyzer at the Functional Genomics Center, University of Rochester, NY. The Gene chip mouse genome 430 2.0 array (Affymetrix) was used to quantify the expression of transcripts and variants from over 34,000 genes. Data were analyzed with Genetraffic (Lobion Informatics Inc.) and Arrayassist Lite software (Stratagene). The genes were considered relevant when their expression was significantly different between WT and TLR4 deficient mice (*P*<.05, Student *t* test). These genes were classified into 2 groups whether they were downregulated 2 fold or more or up-regulated 2 fold or more in TLR4 deficient total liver. Gene expression was further validated with RT-PCR (Taqman, Applied Biosystems), with Addressin-2 (ADSS2) gene expression as a control.

### Depletion of gut-derived bacteria

A broad-spectrum antibiotic cocktail of Ampicillin (1g/L), Vancomycin (500mg/L), Neomycin sulfate (1g/L) and Metronidazole (1g/L) was given in drinking water. The water was replaced every three days for 4 weeks and experiments were performed under aseptic conditions.

### Isolation of granulocytes and enriched fractions of KCs, LSECs and HSCs from the liver

For intrahepatic neutrophil counts, a conventional Percoll gradient was used to harvest total live leukocyte from the liver and neutrophils were characterized as CD11b+ Gr1^high^ TCR- cells. We set up an innovative technique to harvest enriched fractions of KCs, LSECs and HSCs from the same liver. Circulating cells were eliminated by *in situ* perfusion through the portal vein with Ca^2+^-and Mg^2+^-free HBSS supplemented with 5 mM HEPES and 0.5 mM EDTA at 37°C at a flow rate of 4 mL/min. The liver was then perfused with 0.05% collagenase IV (Sigma) buffered with 5 mM HEPES and 0.5 mM CaCl_2_ at 37°C, excised and homogenized. The suspension was washed with RPMI 5% FBS, passed through a 70-μm filter and the filtrate was centrifuged at 50 *g* for 2 minutes to separate hepatocytes (pellet) from the NPCs (supernatant). The NPC-fraction was then submitted to a discontinuous 11.5%-15% Optiprep density gradient, centrifuged at 1500 *g* for 20 min at 4°C for two intermediate layers enriched in KCs and HSCs, but still contaminated with LSECs. For HSCs, KCs and LSECs, both layers were harvested and stained with rat anti-mouse mAbs: FITC-conjugated anti-CD146 (Miltenyi), PE-conjugated anti-Tie2 and APC-conjugated anti-F4/80 (Ebiosciences), APC-Cy7-conjugated anti-CD11b and Pacific Blue live/dead marker (BD Biosciences). LSECs were positively sorted as CD146+ Tie2^high^ (but F4/80- CD11b- CD3-) cells and KCs as F4/80+ CD11b+ (but CD146- Tie2- CD3-) cells. Thus, HSCs were negatively sorted as CD3- CD146- Tie2- F4/80- CD11b- cells. Cell sorting was performed with a 12-colors FACS Aria II (BD Biosciences). The cell purity has been tested with a 4-laser flow cytometer, using 10 parameters. More precisely, after the cell sorting, in a second step, the sorted cells were analyzed with the specific cell markers used for cell sorting. For example, for LSECs isolation, 95% of cells where CD146+ Tie-2^high^ F4/80- CD11b- CD3- cells after cell sorting. All experiments were performed with 95% yield of purity for each subset.

### *In-situ* immunofluorescent detection

The liver from C57BL/6 mice were dissected and fixed with 4% paraformaldehyde in phosphate buffer (pH 7.4) at 4°C, overnight. The tissue was then cryoprotected with series of sucrose gradient (10%, 20% and 30% w/w) in phosphate buffer at 4°C and cut into 20-μm sections. The sections were washed 3 times in phosphate-buffered saline (PBS), blocked in PBS containing 5% goat serum, 1 mg/mL bovine serum albumin (BSA) and 0.1% Triton X-100 for 1 hour at room temperature and incubated with monoclonal rat anti-CXCL1 (1:100; clone: 124014, R&D), polyclonal rabbit anti-CRBP1 (1:50; FL-135, Santa Cruz) or Alexa Fluor 647-conjugated rat anti-F4/80 antibody (Serotec) in PBS containing 1mg/mL BSA and 0.1% Triton X-100, at 4°C, overnight. The sections were washed and stained with Alexa Fluor 546-conjugated goat anti-rat IgG (for CXCL1) or Alexa Fluor 488-conjugated goat anti-rabbit IgG (for CRBP1) (1:500; Invitrogen). In genetically modified mice, GFP expression under Tie-2 promoter restricted GFP signal to LSECs. Sections were stained with TO-PRO-3 (1:1000; Invitrogen) for nuclear visualization in HSCs. All sections were mounted with SlowFade Gold antifade reagent (Invitrogen) and observed with a Leica SL confocal microscope under a 100X objective lens (Leica Microsystems) located in the Keck Imaging Facility at the University of Washington.

### Measurement of cytokine secretion by hepatocytes, KCs, LSECs and HSCs originating from the same liver

Enriched fractions of freshly isolated hepatocytes, KCs, LSECs and HSCs were activated *in vitro* with LPS (1 ng/mL and 100 ng/mL, as a positive control) for 24 hours. First, we used the ELISA (R&D) for the quantification of CXCL1 secretion in the supernatant. Then, a 13-plex multiplex assay was customized to quantify the secretion of TNF, IL-6, IL-1a, IL-10, CCL2, CCL3, CCL4, CCL5, CXCL2, CXCL9, CXCL10, CXCL12 and IFN-g (Bioplex, Biolegend) and data were analyzed using the Luminex XMAP technology (Bioplex software 3.0).

### Measurement of eutrophil migration in response to hepatic stellate cell signal

Neutrophil chemotaxis was evaluated using the Transwell system (5um pore size, Corning Costar). At day 0, freshly isolated WT or TLR4 deficient HSCs were cultured in the lower chamber in complete RPMI overnight, washed and stimulated with 1 ng/mL LPS for 24 hours. At day 2, bone-marrow neutrophils were isolated from WT mice using a conventional Percoll gradient, followed by B- and T-cell MACS depletion (Miltenyi). We added 1.5 X10^6^ neutrophils in 150 μL RPMI supplemented with 20 mM HEPES and 1% fetal calf serum (Perbio Sciences) to the upper chamber and incubated at 37°C for 30 minutes. A sample of 1.5 X10^6^ neutrophils was used as input control (N = 1). To block CXCL1, a monoclonal anti-CXCL1 (R&D) was added to the lower chamber before adding neutrophils. In controls, CXCL1 protein was added to the TLR4 deficient HSCs (100 ng/mL) or used alone (1 ug/mL) as a positive control (N = 1). Neutrophils were harvested in the lower chamber, analyzed using an LSRII flow cytometer (BD Biosciences). Data were analyzed with Flowjo (Treestar) and results were expressed as the percentage of cells that had migrated to the lower chamber.

### Statistical analysis

Data are expressed as mean ± SEM. Quantitative data were compared using non-parametric Mann-Whitney tests and Kruskall Wallis variance analysis. Multiple comparisons were performed using Fisher-PLSD test. *P* values less than .05 were considered significant.

## Results

### CXCL1, the candidate for TLR4-dependent liver crosstalk with gut microbiota

**Fig 1:**
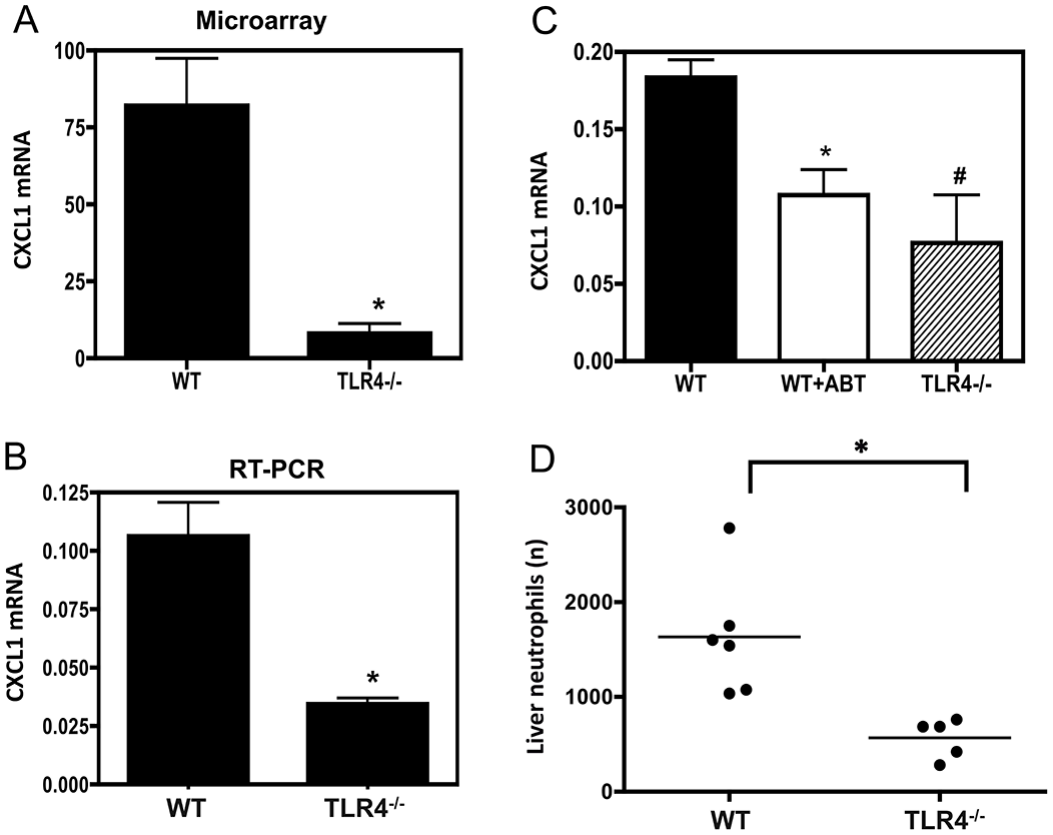
Decrease of CXCL1 message and neutrophil counts in TLR4 deficient liver and after antibiotic treatment. **(A)** CXCL1 expression in the total liver as analyzed by microarrays. Mean values were obtained from three Genechips for three WT and three TLR4 deficient mice. Statistically significant differences between WT and TLR4 deficient mice are indicated by an asterisk, *P<.05, Student *t* test. (**B)** CXCL1 expression measured by quantitative RT-PCR. The relative quantity of CXCL1 mRNA in the total liver of WT and TLR4 deficient mice is indicated (*P<.01). **(C)** Relative expression of CXCL1 in the liver from untreated or antibiotic-treated (ABT) WT mice and TLR4 deficient mice; *P<.01 **(D)** Neutrophils counts in the total liver. CD11+ Gr1^high^ TCR- cells among total live leukocytes isolated from WT and TLR4 deficient liver. In Fig 1B, 1C and 1D, data are representative of five separate experiments with six WT mice (treated or not with antibiotics) and five TLR4 mice; ^**#**^*P*<.05; unpaired Mann -Whitney test.

To document the influence of bacterial LPS on the liver at steady state, we measured the changes in the total gene expression in the LPS-unresponsive liver. Our expression array analysis revealed several genes that were differentially expressed in the liver of WT and TLR4 deficient mice. We focused on genes down-regulated 2 fold or more (Table 1) and genes up-regulated 2 fold or more (Table 2) in TLR4 deficient liver. The gene encoding CXCL1 showed the highest fold difference (10 fold) in the array (Fig 1A), while no other chemokine genes was significantly affected. Hence CXCL1 became the primary focus of our studies. Quantitative RT-PCR confirmed that CXCL1 was significantly decreased in TLR4 deficient liver indicating that CXCL1 expression was consistently lower in the liver of mice unresponsive to bacterial LPS (Fig 1B).

**Table 1:** Genes down-regulated 2-fold or more in the total liver of TLR4 deficient mice compared to WT mice.

Gene ID	Name	Fold increase (*P* value)	Functions
**Npas2**	Neuronal Pas Domain protein-2	19.4 (0.006)	Circadian sleep wake cycle/ locomotive rythm, regulation of transcription
**Cxcl1**	**Chemokine CXC ligand 1 (IL-8)**	**10.2 (0.008)**	**Neutrophil chemoattractant**
**Serpinb1a**	Serine (or cysteine) proteinase inhibitor, clade B, member 1a	5.2 (0.032)	Protein catabolism, peptidase and endo-peptidase inhibitor
**H2Eb1**	Histocompatibility 2, class II antigen E beta	4.3 (0.031)	Antigen presentation
**BirC6**	Baculoviral IAP repeat-containing 6	3.9 (0.048)	Protein ubiquitination, anti-apoptotic
**Btbd5**	BTB (POZ) domain containing 5	3.6 (0.029)	Protein binding
**Mrg1**	Myeloid ecotropic viral integration site-related gene 1	3.8 (0.047)	Eye morphogenesis, regulation of transcription, DNA dependent
**Dnm1l**	Dynamin 1-like	3.4 (0.001)	GTP binding,
**Hba-a1**	Hemoglobin alpha adult chain-1	3.3 (0.036)	Oxygen transport
**Rnd1**	Rho family GTPase 1	3.2 (0.002)	Actin filament organization, negative regulation of cell adhesion
**Zfp101**	Zinc finger protein 101	3.0 (0.035)	Protein coding
**Cyp2b20**	Cytochrome P450, family 2, subfamily b, polypeptide 20	2.8 (0.027)	Electron transport
**Nisch**	Nischarin	2.7 (0.001)	RAC protein, actin cytoskeleton, negative regulation of cell migration
**Hbb-y**	Hemoglobin Y beta like embryonic chain	2.6 (0.028)	Oxygen transport
**Slc25a30**	Solute carrier family 25, member 30	2.5 (0.011)	Transport
**Cd36**	CD36 antigen	2.5 (0.017)	Cell adhesion, receptor activity, protein binding
**Ccng2**	Cyclin G2	2.4 (0.014)	Regulation of cell cycle,
**Rnf125**	Ring finger protein 125	2.3 (0.020)	
**Pde4b**	Phosphodiesterase 4b camp specific	2.2 (0.04)	cAMP specific phosphodiesterase
**H2-Aa**	Histocompatibility 2, class II antigen A, alpha	2.2 (0.044)	Antigen presentation

**Table 2:** Genes up-regulated 2 fold or more in the total liver of TLR4 deficient mice compared to WT mice.

Gene ID	Name	Fold increase (*P* value)	Functions
**Usp2**	Ubiquitin specific protease 2	11.9 (0.012)	Ubiquitin thiolesterase
**LipG**	Lipase, endothelial	5.0 (0.016)	Lipid catabolism
**Ccrn41**	CCR4 carbon catabolite repression 4-like (Nocturin)	5.0 (0.002)	Circadian deadenylase, hepatic steatosis
**Sla**	SRC-like adaptor	4.6 (0.025)	TCR and BCR intracellular signaling cascade
**Per 2**	Period homolog 2	3.3 (0.028)	Circadian rhythm
**Wee1**	Wee 1 homolog	3.1 (0.041)	Cell cycle, serine threonine kinase, Circadian rythm.
**Fdps**	Farnesyl diphosphate synthetase	2.9 (0.006)	Lipid/Steroid biosynthetic process
**Cyp27a1**	Cytochrome P450, family 27, a1	2.8 (0.004)	Hepatic bile acid, fatty acid and cholesterol metabolism
**Rian**	RNA imprinted and accumulated in nucleus	2.8 (0.003)	
**Krt1-13**	Keratin complex 1, acidic, gene 13	2.9 (0.015)	Cytoskeletal organization and biosynthesis
**Sema3e**	Semaphorin domain, secreted 3E	2.9 (0.022)	Interacts with receptor PlexinD1, endothelial cell positioning and patterning of the developing vasculature
**Cklfs2a**	Chemokine-like factor 2A	2.2 (0.005)	Chemotaxis, cytokine activity

In order to assess the functional role of gut bacteria on CXCL1 gene expression in *vivo*, we treated WT mice with oral antibiotic treatment. Liver CXCL1 expression was significantly reduced in antibiotic-treated mice (Fig 1C), confirming that liver CXCL1 expression depends on gut bacteria. Moreover, treating mice with antibiotics mimicked the phenotype observed in TLR4 deficient mice. These data show that gut microbiota influences the baseline expression of one unique chemokine CXCL1 in the healthy liver. Since CXCL1 is a well-established chemoattractant for neutrophils, we tested whether the decrease in CXCL1 in TLR4 deficient liver could modulate neutrophil numbers *in vivo*. The significant role of CXCL1 was confirmed by the decrease of neutrophil counts in the liver of TLR4 deficient mice (Fig 1D).

### Hepatic stellate cells sense low levels of LPS and secrete predominantly CXCL1

**Fig 2:**
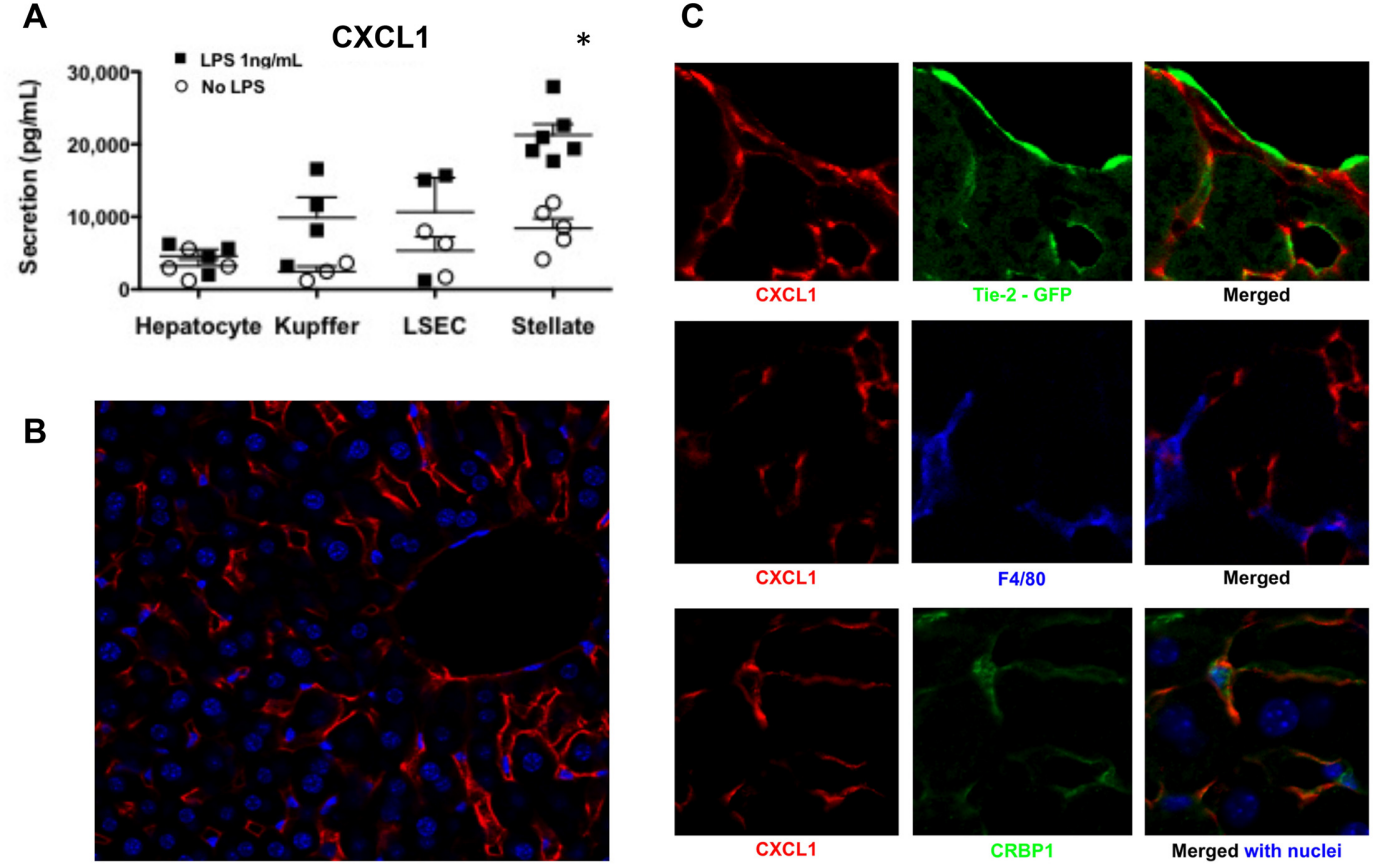
Hepatic stellate cells are the major source of CXCL1, as shown by both quantification of secretion and *in situ* localization. **(A)** Quantification of CXCL1 secretion in enriched fractions of hepatocytes, KCs, LSECs and HSCs, freshly isolated and stimulated *in vitro* with LPS (1 ng/mL LPS, black squares) during 24 hours. Data are representative of three separate experiments with six mice in each group; ^**#**^*P*<.05. **(B)** *In-situ* localization of CXCL1 in the liver. Immunofluorescent detection for CXCL1 (red) and liver cells nuclei (blue) for nuclei first shows CXCL1 expression in the sinusoids throughout liver parenchyma. **(C)** Higher resolution shows that CXCL1 (red) is expressed by sub-endothelial cells, which also store retinol droplets in separate compartments, as shown by CRBP1 staining (green). The Cellular Retinol Binding Protein-1 (CRBP-1) is the best marker to detect simultaneously both resting (Glial Fibrillary Acidic Protein, GFAP+) and activated (α-Smooth Muscle Actin, αSMA+) stellate cells *in situ*. Alexa Fluor-546-CXCL1 (red) staining does not colocalize either with Tie2-GFP in LSECs (green, *upper panel*), or F4/80 in KCs (blue, *middle panel*), but with AlexaFluor-488-CRBP1 (green, *lower panel*), staining both resting and activated HSCs. TOPRO3 was used for nuclei vizualisation.

To identify the source of CXCL1 in the liver, we set up an innovative technique based on cell sorting to harvest enriched fractions of KCs, LSECs and HSCs from the same liver, to a yield of 95% purity. In a first set of experiments, we quantified CXCL1 secretion in liver cells freshly isolated from WT mice, followed by *in vitro* stimulation with or without low levels of LPS (1 ng/mL). CXCL1 was mostly secreted by HSCs, followed by LSECs and KCs, whereas hepatocytes were the poorest producers of CXCL1 (Fig 2A).

To confirm the source of CXCL1 *in situ*, we performed immunofluorescent staining with specific markers for liver cells at steady state. This alternative approach avoided the complication that cytokine expression could have been modified after a lengthy process of isolation and cell sorting. In the normal liver, CXCL1 expression had a clear sub-endothelial localization as shown by Tie-2 staining (Fig 2B, *upper panel*), confirming the close relationship to LSECs. The CXCL1 expression did not co-localize with F4/80 expression, excluding the detection of CXCL1 in Kupffer cells (*middle panel*). CXCL1 and cytoplasmic CRBP1 were both localized in star-shaped cells, the stellate cells (*lower panel*). The Cellular Retinol Binding Protein-1 (CRBP-1) was the best marker to detect simultaneously both resting (Glial Fibrillary Acidic Protein, GFAP+) and activated (α-Smooth Muscle Actin, αSMA+) stellate cells *in situ*. Taken together with the secretion assay performed on isolated cells, these data confirm that the HSCs are the major source of the neutrophil chemo-attractant CXCL1 in the liver.

### Hepatic stellate cells have a secretion profile different form other cells in response to low levels of endotoxins

**Fig 3:**
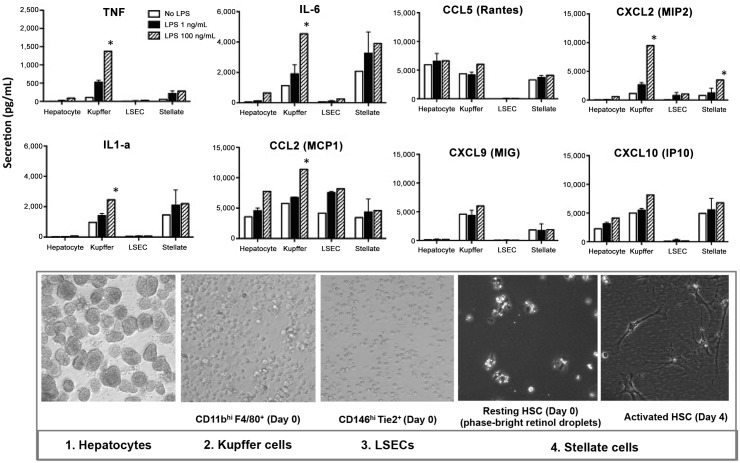
Cytokine secretion by hepatocytes, KCs, LSECs and HSCs after isolation from the same liver and in response to low levels of LPS. Liver cells were freshly isolated on density gradient followed by cell sorting and stimulated with LPS (1ng/mL LPS, black bars or 100ng/mL LPS, hatched bars). Cytokine secretion was measured in the same supernatant with a multiplex assay, run in triplicates. Graphs show three experiments with six mice in each group and statistically significant differences (*P<.05) between basal LPS stimulation (1ng/mL) and higher LPS stimulation (100ng/mL) are indicated. Lower panel: bright field images of cells right after isolation (Hepatocytes, LSECs, KCs). Images of HSCs at higher resolution show the retinol droplets at Day 0 and the typical shape of the activated stellate cells after 4 days in culture.

In a different set of experiments, we investigated the cytokine secretion of the enriched fractions of KCs, LSECs and HSCs from the same liver, using a 13-parameter multiplex assay. Interestingly, all liver cells were able to respond to levels of LPS as low as 1 ng/mL (Fig 3). The secretion of all cytokines increased with a higher concentration of LPS, 100 ng/mL. The only chemokine secreted in large amounts by all liver cells was CCL2, with concentrations ranging from 4560 pg/mL to 7560 pg/mL. As expected, TNF, CXCL2 and CXCL9 were almost exclusively secreted by KCs (520 pg/mL, 2670 pg/mL and 4360 pg/mL, respectively). IL-6 and IL-1 were secreted exclusively by KCs and HSCs. Conversely, IL-10, CXCL12, CCL3, CCL4 and IFN-gamma secretion levels were very low or undetectable (data not shown). In conclusion, we show that HSC are the major source of CXCL1 in the liver and—while HSC secretion profile is similar to Kupffer cells regarding secretion of most cytokines-, it was unexpected that the unstimulated hepatic stellate cells are able to secrete as high amounts of CXCL1 as Kupffer cells or LSECs, stimulated with low levels of LPS.

### Neutrophil migration requires CXCL1 secretion by hepatic stellate cells following basal TLR4 activation

**Fig 4:**
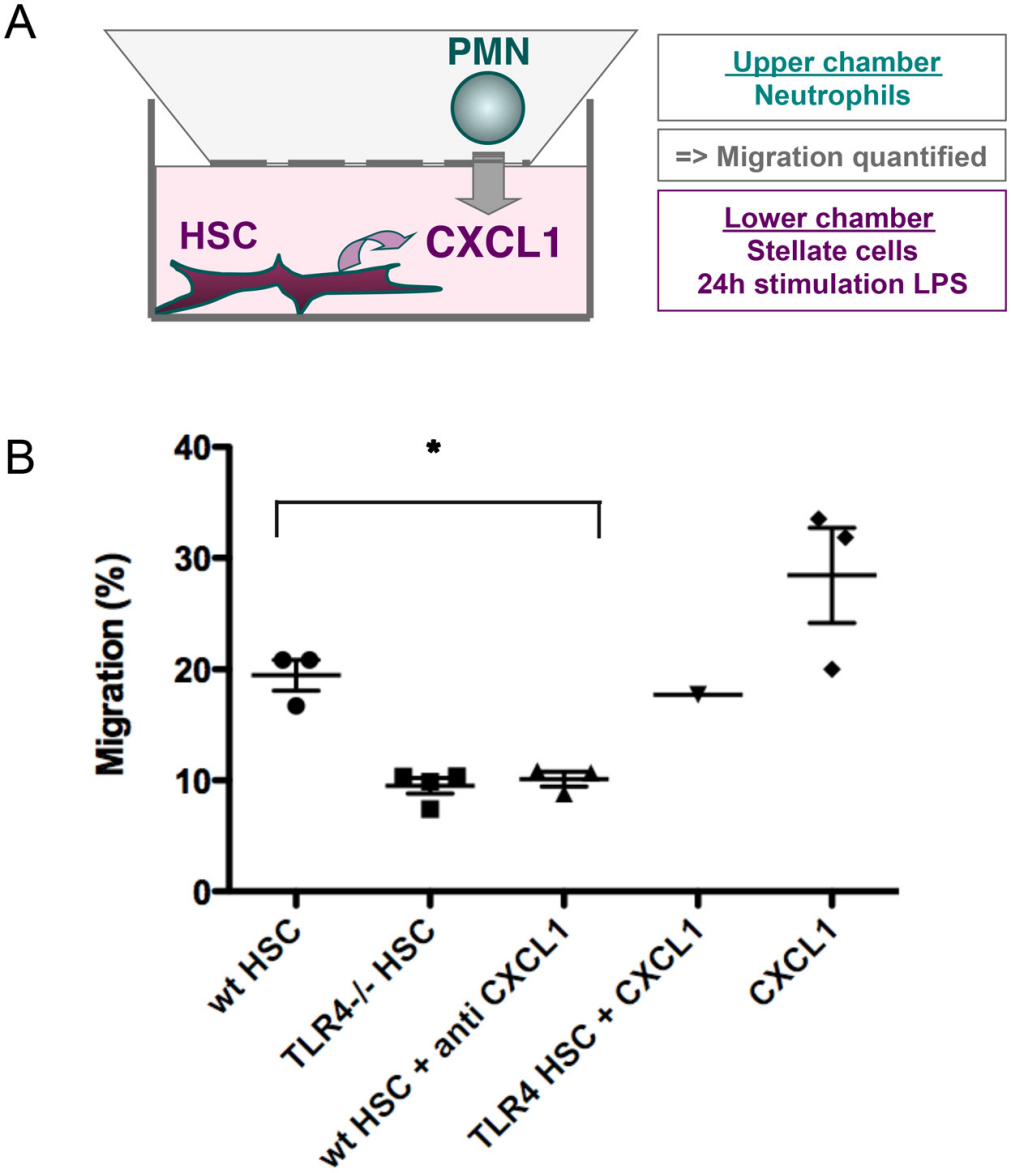
Neutrophils migrate in response to CXCL1 secretion following TLR4 activation in hepatic stellate cells. **(A)** Schematic representation of the neutrophil chemotaxis assay. **(B)** Quantification of neutrophil migration in response to secretory WT or TLR4 deficient stellate cells. WT stellate cells were treated (WT HSC + anti CXCL1) or not with anti-CXCL1 antibody. As for internal positive control, the migration of neutrophils towards TLR4 deficient stellate cells supplemented with CXCL1 protein (TLR4 HSC + CXCL1) and with CXCL1 protein only (CXCL1) was quantified in only one experiment. Graphs show three experiments with six mice in each group and statistically significant differences (*P<.05) between WT HSCs and TLR4 deficient HSCs, as well as between WT HSCs treated or not with anti-CXCL1, are indicated.

Since CXCL1 is a recognized chemoattractant for neutrophils, we tested whether CXCL1 secreted by hepatic stellate cells was able to trigger a functional chemotactic effect on bone marrow derived neutrophils. Neutrophils migration was quantified following basal TLR4 activation in HSCs (Fig 4A). Briefly, at day 0, freshly isolated stellate cells from WT or TLR4 deficient mice were stimulated with 1ng/mL LPS for 24 hours in lower chamber. At day 2, bone marrow derived neutrophils were loaded to the upper chamber and their migration towards secretory stellate cells was analyzed. Neutrophils migrated less toward TLR4 deficient HSCs than towards WT HSCs. The addition of anti-CXCL1 blocking antibody to the lower chamber mimicked the migration of neutrophils towards TLR4 deficient HSCs (Fig 4B). In one of the experiments, the addition of the recombinant CXCL1 protein to TLR4 deficient HSCs restored neutrophil migration to the level towards WT stellate cells, and was used as an internal control. These data show for the first time that the CXCL1 secretion by stellate cells in response to low levels of LPS is dependent on the specific activation of TLR4, and exerts its well known functional chemotactic activity on neutrophils.

Taken together, these data support a model in which microbial products act on HSCs *via* TLR4 for the secretion of a functional CXCL1- as shown by neutrophil chemotaxis. Both *in situ* and *in vitro* data converge to the conclusion that the secretion of CXCL1 (the homologue of human IL-8) by hepatic stellate cells is a key component of the liver’s response to gut microbiota at steady state.

## Discussion

In this study, we present new evidence that HSCs are of central importance in response to low levels of bacterial products, due to their secretion of the chemokine CXCL1 / IL-8. In recent years, the direct role of gut bacteria in liver homeostasis is an emerging concept.[8] As in the intestine, the activation of TLRs in the liver could provide information about the bacterial load in the portal blood, and activate the synthesis of antimicrobial proteins in order to maintain the surface-associated bacterial population at homeostatic levels.[18] After the recent publications showing that not only LPS but also other ligands for TLRs such as DAMPs could play a role in the liver,[19] we herein show the specific and essential activation of TLR4 in the liver cells. The influence of gut microbiota on liver immunology has been mostly explored in the context of inflammatory, toxic or metabolic liver disease.[8, 20] Here, in contrast, we study the steady state liver and focus our experiments on low concentrations of LPS found in the incoming portal blood draining the liver. We isolated enriched fractions of hepatocytes and non-parenchymal cell populations and were able to compare for the first time the specific cytokine/chemokine secretion profile of KCs, LSECs and HSCs isolated from the same liver. Notably CCL2, a well-known chemoattractant for monocytes/macrophages, is the chemokine secreted in large amounts by all these cell types, all responding to low levels of LPS. Our study highlights the complexity of the crosstalk between the liver cells and we hypothesize that monocyte/macrophage homing might also be regulated *via* TLR4 signaling in a normal liver.[2]

Although all liver cells express TLR4, the hepatic stellate cells are the critical population that promotes fibrosis in a TLR4-dependent manner once activated.[10] Both quiescent and activated HSCs express high levels of TLR4 and LPS directly targets HSCs *in vivo*.[21] TLR4-dependent mechanisms occur specifically in HSCs in interaction with LSECs during vascular remodeling in fibrosis or in early phases of hepatocellular carcinoma promotion, making TLR4 a potential therapeutic target for prevention in advanced liver disease.[22, 23] In the present study, stellate cells are cultured for 24 hours, which was not long enough to induce their trans-differentiation; therefore we argue that the cells in our cultures are reproducing their biology in the liver *in vivo*.

The microarray analysis—performed in total liver—identified a number of genes that were differentially expressed in the WT and TLR4 deficient tissue. Among genes with a known immunologic function, CXCL1 showed the highest fold difference and was therefore the principal candidate for an effect of TLR4 signaling on liver immune function. LPS is a strong inducer of this chemokine and possible sources for CXCL1 in the liver were hepatocytes, KCs and HSCs [24, 25, 26]. CINC, the rat homolog for CXCL1, was detected in HSCs [27]. Here, going further down to the cellular level, we manage to show that the HSCs are the major source of CXCL1 in the mouse liver, in response to low levels of LPS.

We found that neutrophil counts were decreased in TLR4 deficient liver. Migration assays showed that neutrophils migrated less towards HSCs isolated from TLR4 deficient liver. Thus, TLR4 signaling in HSCs is necessary for neutrophil recruitment. In pathology, overexpression of CXCL1 and increased neutrophil infiltration has been studied in inflammatory liver injury and in other organs.[28] Intrahepatic neutrophils produce molecules that can attract additional neutrophils, T cells and macrophages. Conversely, in a model of peritonitis-induced sepsis, early and local treatment with CXC chemokines enhances neutrophil recruitment and bacterial clearance.[29] In ischemia-reperfusion injury, neutrophil play a protective role through the induction of an oxidative burst, which leads both to increased antimicrobial activity and the intestinal epithelial barrier integrity.[30] Similarly, in the normal liver, the antibacterial function of neutrophils could protect liver integrity from low-level bacterial translocation.

The mechanism we have clarified here enhances the understanding of the relationship between the resident microbiota and the immune balance of the liver, which plays an important part in the regulation of liver fibrosis. In steady state, low levels of bacterial products constitutively pass from the gut to the liver, signal through TLR4, and hepatic stellate cells secrete CXCL1. During fibrosis, if HSCs sense additional danger signals, their activation results in liver cytokine secretion imbalance, together with ongoing trans-differentiation. Direct evidence for such a mechanism would suggest it might be possible to intervene therapeutically to favor the resolution of fibrosis. This is a fertile area for further research.
